# The role of monocytes and macrophages in idiopathic inflammatory myopathies: insights into pathogenesis and potential targets

**DOI:** 10.3389/fimmu.2025.1567833

**Published:** 2025-03-20

**Authors:** Shinji Izuka, Toshihiko Komai, Yumi Tsuchida, Haruka Tsuchiya, Tomohisa Okamura, Keishi Fujio

**Affiliations:** ^1^ Department of Allergy and Rheumatology, Graduate School of Medicine, The University of Tokyo, Tokyo, Japan; ^2^ Department of Functional Genomics and Immunological Diseases, Graduate School of Medicine, The University of Tokyo, Tokyo, Japan

**Keywords:** idiopathic inflammatory myopathy, myositis, dermatomyositis, monocytes, macrophages, interstitial lung disease, interferon, mitochondrial dysfunction

## Abstract

Idiopathic inflammatory myopathies (IIMs) are heterogeneous autoimmune disorders characterized by muscle inflammation, weakness, and extramuscular manifestations such as interstitial lung disease, skin rash, arthritis, dysphagia, myocarditis and other systemic organ involvement. Although T and B cells have historically been central to the understanding of IIM immunopathology, monocytes and their differentiated progenitor cells, macrophages, are increasingly being recognized as critical mediators of both tissue damage and repair. In subtypes such as dermatomyositis, immune-mediated necrotizing myopathy and antisynthetase syndrome, macrophages infiltrate skeletal muscle and other affected tissues, contributing to inflammation via production of pro-inflammatory cytokines, chemokines, and reactive oxygen species. Dysregulated interferon signaling, mitochondrial stress, and aberrant metabolic states in these cells further perpetuate tissue injury in IIMs. Conversely, certain macrophage subsets can support muscle fiber regeneration and dampen inflammation, underscoring the dual roles these cells can play. Future research into the heterogeneity of monocytes and macrophages, including single-cell transcriptomic and metabolomic approaches, will help clarify disease mechanisms, identify biomarkers of disease activity and prognosis, and guide novel therapeutic strategies targeting these innate immune cells in IIM.

## Introduction

Idiopathic inflammatory myopathies (IIMs) represent a diverse group of autoimmune diseases characterized by immune-mediated damage to skeletal muscle. IIM subgroups include dermatomyositis (DM), antisynthetase syndrome (ASyS), polymyositis (PM), immune-mediated necrotizing myopathy (IMNM), and inclusion body myositis, each of which exhibits distinct clinical and pathological features (1). IIM manifestations extend beyond muscle weakness, frequently involving skin rashes, arthritis/arthralgia, interstitial lung disease (ILD), and cardiac complications (1–3). While early research focused primarily on the roles of T and B lymphocytes and dendritic cells, a growing body of evidence highlights the crucial involvement of monocytes and macrophages in the initiation, progression and resolution of muscle inflammation in IIMs (4–7). Monocytes are recruited to affected tissues where they can be activated, differentiating into various forms of macrophages that contribute to tissue damage, modulate the local inflammatory milieu and participate in repair mechanisms. Given the increasing recognition of the roles of monocytes and macrophages in IIM pathology, this review aims to delineate their diverse functions, explore their contribution to disease mechanisms, and discuss potential targeted therapeutic strategies.

## Monocyte-to-macrophage differentiation and polarization

The myeloid cell compartment, comprising monocytes, macrophages, granulocytes and dendritic cells, serves not only as the first line of immune defense but also plays a pivotal role in mediating communication between innate and adaptive immunity (8, 9). Traditional flow cytometry methods for classifying blood monocytes have relied on the distinction among classical (CD14^++^CD16^−^), intermediate (CD14^++^CD16^+^), and non-classical (CD14^+^CD16^++^) subsets (10). However, single-cell RNA sequencing (scRNA-seq) has revolutionized our understanding of monocyte heterogeneity, revealing a more complex and dynamic picture of their subsets. This technology demonstrates a spectrum of transcriptional states within these subsets, each characterized by specific cytokine profiles, interferon (IFN) signatures, chemokine receptor expression and metabolic activity (11, 12). This refined understanding will be crucial for dissecting the specific roles of different monocyte and macrophage populations in the pathogenesis of IIMs.

While not all macrophages are derived from monocytes (13), in the context of myositis, monocytes are recruited to affected muscle tissues, where they differentiate into monocyte-derived macrophages (14). Once within the muscle tissue, these monocytes undergo further differentiation into functionally plastic macrophages, exhibiting a wide range of activation states (15). Macrophages are traditionally categorized as M1-like (pro-inflammatory) or M2-like (anti-inflammatory and tissue-repairing) subsets; however, *in vivo* macrophage phenotypes are considerably more nuanced and context-dependent (16). Indeed, macrophages exhibit pro-inflammatory, pro-wound-healing, pro-fibrotic, anti-inflammatory, anti-fibrotic, pro-resolving, and tissue-regenerating properties, often with overlapping or sequential expression (16). M1-like macrophages are often activated by IFN-γ and bacterial products via pathways including the IFN-γ/Janus kinase (JAK)/signal transducer and activator of transcription 1 (STAT1), Toll-like receptor (TLR) activation of TIR domain-containing adaptor inducing interferon-β/interferon regulatory factor 3 for IFN secretion, and TLR/MyD88/NF-κB for cytokine secretion, and these macrophages secrete inflammatory cytokines and chemokines (e.g., tumor necrosis factor (TNF)-α, interleukins (IL-1, IL-6), chemokines (e.g., C-C motif ligand (CCL)2/4, and C-X-C motif chemokine ligand (CXCL)8/11), nitric oxide, and reactive oxygen species (ROS) (15). These factors exacerbate local inflammation and contribute to muscle fiber necrosis.

Macrophages also play a crucial role in resolving inflammation by clearing cellular debris. M2-like macrophages, often induced by IL-4 and IL-10 via the IL-4/IL-13/JAK1/JAK3/STAT6 and IL-10/STAT3 pathways, respectively, secrete anti-inflammatory mediators (e.g., IL-10) and the growth factors IGF-1 and transforming growth factor β, which can both promote tissue repair and, in some contexts, contribute to fibrosis (17). They may also promote myogenic differentiation and facilitate muscle fiber regeneration. However, the M1/M2 paradigm should be considered a spectrum, with considerable plasticity in macrophage phenotypes influenced by the local microenvironment (16). Understanding these diverse macrophage phenotypes lays the groundwork for examining changes in circulating monocyte subsets and their contribution to the tissue-specific pathology of IIMs.

## Circulating monocytes in myositis

Studies of circulating monocyte populations in IIMs have revealed alterations in subset distribution and activation status. Notably, in patients with active anti-melanoma differentiation-associated protein 5 (MDA5) antibody-positive DM, the proportion of circulating classical monocytes was increased compared with healthy controls (18). Classical monocytes are characterized by their production of S100 family proteins, leading to pro-inflammatory responses (19, 20). Furthermore, monocyte subsets in IIM, including classical monocytes, show elevated expression of genes involved in neutrophil and monocyte activation, such as *S100A8, S100A9, S100A12*, *FCGR3B*, and *CXCR2* (18). Similarly, mass cytometry of peripheral blood mononuclear cells (PBMCs) revealed an increased proportion of CD14^+^CD16^-^CD19^-^CD3^-^ classical monocytes in IIM patients compared with healthy controls (21). Furthermore, bulk RNA-seq analysis of PBMCs from patients with IIMs, along with deconvolution analysis using CIBERSORTx algorithm (22), also indicates an increased proportion of monocytes compared to healthy controls (23). In the study, flow cytometry analysis further confirmed the increased proportions of classical and intermediate monocyte subsets in IIM patients compared to healthy controls. These changes in PBMC populations have been identified in patients with both anti-MDA5 antibody-positive DM and anti-Jo-1 antibody-positive IIMs (23). Notably, scRNA-seq of PBMCs detected *IFI27*-expressing CD14^+^ monocytes as a key feature in patients with active DM positive for anti-MDA5 antibodies (24). Since *IFI27* is an IFN-inducible gene, this finding suggests that IFN signature and activated monocytes are contributing to the pathogenesis of anti-MDA5 antibody-positive DM.

## Monocyte recruitment and chemokine dynamics in myositis

In the pathogenesis of myositis, monocytes are actively recruited from the circulation to inflamed muscle tissue. This process is driven by intricate interplay among chemokines and adhesion molecules (14). Notably, elevated levels of several chemokines, including CCL2, CCL3, CCL4, CXCL8, CXCL9, and CXCL10, have been identified within the muscle tissue of IIM patients (25–31). These chemokines act as chemoattractants, guiding monocytes and lymphocytes to the site of inflammation (31). Specifically, CCL2, CCL3, CCL4, CXCL8, and CXCL10 levels are also increased in the peripheral blood of patients with IIM (28, 32, 33), suggesting their role in the systemic inflammatory response simultaneously. Moreover, the serum CXCL8 level has been identified as a predictive marker for rapidly progressing ILD (RP-ILD) among patients with IIM-associated ILD (34). Similarly, the serum CXCL10 level is correlated with the Cutaneous Dermatomyositis Disease Area and Severity Index (CDASI) score in patients with DM (35). Further emphasizing this connection, the serum CXCL10 levels in anti-MDA5 antibody-positive DM patients are markedly elevated at disease onset and decrease upon treatment (36). This highlights the systemic nature of inflammation and the involvement of chemokines in multi-organ manifestations of IIMs. Interestingly, monocytes have been shown to produce CXCL10 in a dose-dependent manner upon type I IFN stimulation *in vivo* (36). This suggests a positive feedback loop wherein monocyte activation and type I IFN signaling amplify inflammation. In addition, increased levels of vascular adhesion molecules, such as vascular cell adhesion molecule 1, are also observed in patients with DM, particularly those who develop severe ILD (37). This increased expression of adhesion molecules likely further facilitates the migration of monocytes into the damaged organs.

## Macrophage infiltration of muscle tissue in myositis

Histopathological studies consistently reveal significant infiltration of monocytes and macrophages within the muscle tissue of IIM patients (1, 14). Macrophages are often found distributed around the endomysium and perimysium in patients with DM. However, the distribution of macrophages in affected muscle can vary considerably depending on the IIM subtype and specific autoantibody profile. For instance, muscle specimens from juvenile patients with anti-nuclear matrix protein (NXP) 2 and anti-transcriptional intermediary factor (TIF) 1-γ antibodies show pronounced diffuse endomysial macrophage infiltration (38). Conversely, muscle biopsies from anti-MDA5 antibody-positive DM patients typically show only mild inflammatory cell infiltration (38). However, biopsies of juvenile DM patients with anti-Mi2 antibodies in one study tended to show greater necrosis or perifascicular atrophy rather than purely diffuse macrophage infiltration (39). These infiltrations mirrored those found in adult DM; anti-NXP2, anti-TIF1-γ, and anti-Mi2 antibody-positive DM displayed similar CD68^+^ infiltration in both endomysial and perimysial areas, while anti-MDA5-positive DM showed less CD68^+^ cell infiltration (40) (**Supplementary Figure S1**). In patients with ASyS (41) and IMNM (42), macrophages are diffusely distributed within the endomysium. This implies that the degree and pattern of macrophage infiltration reflect distinct underlying pathogenic mechanisms in different IIM subtypes. Once recruited to muscle tissues, macrophages contribute to myophagocytosis, the process of engulfing damaged muscle fibers, and release pro-inflammatory cytokines, including IL-1, IL-6, and TNF (43). These actions amplify the inflammatory response and directly contribute to muscle damage.

Our previous transcriptomic analysis revealed a strong association between increased monocyte infiltration into muscle tissue and muscle damage (6). By applying a deconvolution algorithm to bulk RNA-seq data from muscle tissue of IIM patients, we quantified the proportions of infiltrating immune cell types. We found that the estimated proportions of CD16^+^ and CD16^-^ monocytes, as well as myeloid dendritic cells, are positively correlated with serum creatine kinase and aldolase levels, further reinforcing their involvement in muscle damage. Similarly, gene modules associated with phagocytosis also exhibit a positive correlation with these muscle enzymes. Supporting these findings, histological analysis of muscle tissue from patients refractory to standard immunosuppressive treatment for myositis revealed a larger area occupied by CD68^+^ macrophages compared with muscle tissue from patients who responded to treatment (44). Additionally, a negative correlation was observed between the manual muscle testing 8 score at baseline and the area occupied by CD68^+^ cells before treatment. This suggests that the extent of macrophage infiltration may be a predictive marker of disease severity and treatment response.

## Monocyte and macrophage contributions to pulmonary manifestations in myositis

While direct evidence of monocyte and/or macrophage infiltration in lung tissues from IIM patients is limited, this is largely because bronchoalveolar lavage fluid (BALF) and lung biopsies are not necessary for clinical diagnosis and do not substantially contribute to clinical decision-making. This is especially true when radiological patterns of ILD are evident through imaging or serological markers such as myositis-specific antibodies or myositis-associated antibodies are positive (45, 46).

Nonetheless, there is growing evidence suggesting that macrophages likely play a significant role in the development of IIM-associated ILD. One immunohistochemical analysis showed that CD163^+^ macrophages were found to infiltrate the alveolar spaces in the lungs of patients with DM-related ILD and were more severe in the lungs of a non-surviving patient (47). The serum level of soluble CD163, a type I transmembrane protein and a marker of macrophage activation, is also elevated in DM and PM patients with ILD compared to those without ILD, and it correlates with disease activity (48). Additionally, recent scRNA-seq of BALF from patients with DM revealed an upregulation of genes associated with IFN-related pathways, including *IFIT1* and *CXCL10*, within monocytes/macrophages (49). Interestingly, a viral response signature, characterized by *MX1* expression, was detected not only in lung-resident immune cells but also in circulating monocytes, neutrophils, B cells, and plasma cells in the blood of DM patients. This suggests a systemic immune response with a prominent pulmonary component in these patients.

Several serum markers associated with macrophage activation have been reported to predict prognosis in IIM-associated ILD. Ferritin is described as a macrophage activation marker and is introduced alongside other macrophage markers such as chitinase-3-like protein 1, soluble CD206, galectin-9, and neopterin in the context of anti-MDA5 antibody-positive DM (50–53). Although the precise cellular origin of ferritin in this condition remains unclear, the positive correlation between an increased percentage of HLA-DR^low^ CD14^+^ monocytes and both serum ferritin and IL-6 levels (54) suggests that these monocytes, in addition to macrophages, may also be a source of ferritin (55). Moreover, macrophages recycle iron from senescent erythrocytes, storing it intracellularly in ferritin. In inflammatory states, an elevated level of hepcidin leads to the degradation of ferroportin, causing macrophages to retain more iron. This results in increased ferritin synthesis and release, thus elevating the serum ferritin concentration. During macrophage activation, pro-inflammatory cytokines (e.g., IL-1β, IL-6) and hepcidin signals converge to upregulate ferritin expression (56). Additionally, the heavy subunits of ferritin may also function as a pro-inflammatory factor (57). While the ferritin level is linked to disease progression, particularly in patients with RP-ILD, the serum CXCL10 level has been reported to reflect the early treatment response more effectively compared with the ferritin level (36). In addition to ferritin, serum soluble CD206, a mannose receptor and marker for M2 macrophages (58) is also elevated in anti-MDA5 antibody-positive DM-associated ILD (59) and in RP-ILD (60). Furthermore, its serum level is correlated with a poor prognosis, indicating a potential pathogenic role (61). Although M2 macrophages generally play an anti-inflammatory role, CD206^+^ lung macrophages in bleomycin-induced lung fibrosis in mice contribute to lung fibrosis, suggesting a pathogenic fibrotic role in IIM-ILD (62). Neopterin, primarily produced by activated macrophages and monocytes stimulated with IFN-γ, exhibits higher serum levels in DM patients compared with healthy controls (63). Anti-MDA5 antibody-positive DM patients exhibit the highest levels of serum neopterin, and a high serum neopterin level was identified as an independent risk factor for mortality (63). Additionally, the serum level of YKL-40, primarily secreted by macrophages and neutrophils, has also been shown to predict the occurrence of RP-ILD and to indicate a poor prognosis (64). These findings underscore the complex interplay among macrophage activation, IFN signaling, and ILD disease severity, though interpretation should be approached with caution given the plasticity and heterogeneity of macrophages.

Emerging evidence suggests that macrophage polarization is not strictly binary (65), particularly in tissue-resident macrophages such as alveolar macrophages (AM). Indeed, AM from individuals in distinct tissue localization consistently express features of both M1 (CD80, CD86, CD64) and M2 (CD206 and CD163) polarized macrophages, and the CD206^hi^CD86^hi^ AM subset remains the dominant population even after *in vivo* exposure to pneumococci or human immunodeficiency virus (66). Additionally, M2 macrophages can express CD86, an M1 marker, in response to TLR2 stimulation and exhibit a pro-inflammatory phenotype, further demonstrating the phenotypic and functional plasticity of macrophages. Moreover, macrophages exhibit significant heterogeneity within the M2 spectrum, encompassing distinct subsets (M2a, M2b, M2c, and M2d) with diverse roles in wound healing, immunoregulation, and tissue remodeling (65, 67). Given this complexity, the relationship between M2 macrophages and M1 markers cannot be simply defined, and macrophage-associated parameters must be interpreted within specific biological contexts.

## Macrophage infiltration of skin in myositis

The histology of DM skin is characterized by perivascular infiltrates of immune cells, including T cells and macrophages (68). Immunohistochemical analysis of DM skin lesions (specifically Gottron’s sign) in patients with anti-MDA5 antibody-positive DM revealed that the majority of CXCL10^+^ cells are also CD68^+^, indicating their monocyte/macrophage lineage (36). Furthermore, in addition to the presence of MxA, a protein specifically induced by type I IFN, CXCL10 is moderately to strongly expressed in DM skin, primarily within the upper dermis and epidermis (69). These skin areas also exhibit elevated numbers of CXC-chemokine receptor 3 (CXCR3)^+^ T cells. This suggests a potential mechanism wherein type I IFN-induced CXCL10 production by macrophages attracts T cells, contributing to the characteristic skin rash in DM.

While IFN signatures in muscle tissue are reportedly enriched in DM and, to a lesser extent, in ASyS (6, 70), studies employing immunofluorescence and highly multiplexed imaging mass cytometry showed that both ASyS and DM skin exhibit similarly elevated type I IFN signaling. This is evidenced by elevated levels of IFN-β and MxA compared with healthy controls (71). Interestingly, macrophage subsets, specifically those positive for phosphorylated stimulator of interferon genes (STING), display heightened production of pro-inflammatory mediators, such as TNF, IL-17, and IFN-β, in skin involvement in ASyS relative to those in DM (71). In contrast, the overall composition and activation of other immune cell populations, including dendritic and T cells, are largely similar between the two diseases. These findings demonstrate that while both ASyS and DM skin share common features of type I IFN activation, macrophages positive for phosphorylated STING may play a distinct role in modulating inflammation and contributing to the unique clinical features of cutaneous signs in ASyS.

## Monocyte activation and mitochondrial dysfunction in myositis

The activation of monocytes in IIMs is influenced by complex interplay among factors such as galectin-9, IFN signaling, and mitochondrial dysfunction. Galectin-9, a β-galactose-binding protein secreted by vascular endothelial cells, promotes angiogenesis (72). In particular, the serum galectin-9 level is significantly increased in patients with anti-MDA5 antibody-positive DM compared with healthy controls (73–75). Furthermore, increased gene expression of galectin-9 is observed in both the serum and lung tissues of patients who developed RP-ILD (75). *In vitro* experiments confirmed that stimulation with galectin-9 leads to increased secretion of CCL2 from lung fibroblasts (75). Given that galectin-9 promotes differentiation into M2-type macrophages (76), these findings suggest that galectin-9 can stimulate monocyte/macrophage activity and contribute to lung fibrosis especially in anti-MDA5 antibody-positive DM. Interestingly, a positive correlation was observed between galectin-9 and type I IFN-inducible genes, such as *MX1* and *IFIH1*, at the mRNA level in PBMCs, suggesting a potential synergistic or interconnected role of galectin-9 and type I IFN signaling in fibrosis associated with anti-MDA5 antibody-positive DM (75). This further emphasizes the intricate interplay of different inflammatory mediators in IIM pathogenesis.

Lately, emerging evidence has also highlighted the contribution of monocyte mitochondrial dysfunction to IIM pathology. In juvenile dermatomyositis (JDM), mitochondrial abnormalities in CD14^+^ monocytes, including the presence of “megamitochondria” and enhanced oxidative phosphorylation, promote the production of oxidized mitochondrial DNA (77). This oxidized mitochondrial DNA can activate the cyclic GMP-AMP synthase/STING pathway, driving further IFN production and muscle fiber damage. Additionally, dysregulated expression of mitochondrial-associated genes has been correlated with increased expression of IFN-stimulated genes in JDM CD14^+^ monocytes, highlighting this mechanistic link (77). Indeed, IFNs activate the transcription factor STAT1, which in turn induces the expression of M1 markers, including CXCL9 and CXCL10, via the IFN-α/β receptor (78, 79). These findings underscore the role of IFN signaling in promoting pro-inflammatory macrophage activation. Moreover, aberrant changes to mitochondrial morphology and cellular metabolism are key features of mitochondrial stress, associated with decreased oxidative phosphorylation and increased ROS levels (15, 80, 81). These observations highlight dysregulated mitochondrial dynamics and oxidative stress as critical pathological features linking monocyte abnormalities to IIM disease progression.

## Crosstalk between monocytes/macrophages, other immune cells, and muscle fibers

Monocytes and macrophages do not function in isolation in IIM; rather, they interact extensively with other immune cells, including CD8^+^ cytotoxic T cells, CD4^+^ helper T cells, and B cells (82). Activated macrophages initiate a cycle of inflammation by releasing pro-inflammatory cytokines and chemokines. These signals attract more immune cells, activate helper and cytotoxic T cells, which leads to tissue damage, and stimulate further macrophage activity. C-X3-C motif ligand 1 (CX3CL1), which is highly expressed in inflamed endothelial cells induced by type I IFN (83), plays a crucial role in attracting monocytes and T cells. An elevated CX3CL1 level in patients with anti-MDA5 antibody-positive DM shows a significant correlation with anti-MDA5 antibody titers (84). Notably, CX3CL1 can induce recruitment of CX3CR1^+^ M2 macrophages in the lungs (83, 85), potentially associated with lung fibrosis, suggesting complex interplay among endothelial activation, monocyte recruitment, and tissue fibrosis. In addition, dysregulation of regulatory T cells, which normally regulate macrophage phagocytosis, can contribute to disease development (86). These intricate cellular interactions might be critical to the pathogenesis of muscle damage. Unlike in healthy conditions, muscle fibers themselves can produce chemokines and express MHC class I in IIMs (82, 87), resulting in dynamic interplay in which both immune and muscle cells influence the other’s phenotypes and activities. Furthermore, monocyte-derived dendritic cells can facilitate myoblast proliferation and migration while upregulating the expression of HLA-ABC, HLA-DR, VLA-5, and VLA-6 in myoblasts (88). This further indicates that monocyte-derived cells directly influence muscle regeneration and repair processes in IIMs.

## Therapeutic implications

A deeper understanding of the multifaceted roles of monocytes and macrophages in IIM pathogenesis is paving the way for the development of more targeted therapeutic strategies. However, the current therapies targeting these cells in IIMs are not fully optimized. The development of treatments that directly address monocyte recruitment, modulate their activation, shift their polarization states, or address the underlying mechanisms of their aberrant activation (e.g., by addressing mitochondrial dysfunction or aberrant type I IFN signaling) are active and important areas of investigation.

## Effect of current standard therapies for IIMs on monocyte/macrophages

Current standard of care treatments for IIM, including glucocorticoids, methotrexate, azathioprine, calcineurin inhibitors, cyclophosphamide and intravenous immunoglobulin (IVIg) (1, 89), likely exert some of their therapeutic effects by modulating monocyte and macrophage function, although the precise mechanisms are not fully elucidated. Glucocorticoids can directly and indirectly influence immune cell differentiation, exerting distinct effects on monocytes versus mature macrophages. Indeed, they alter a greater number of mRNAs in monocytes than in differentiated macrophages (90) and can inhibit proinflammatory mediators while enhancing anti-inflammatory factors, partly by stimulating the adenosine receptor A3 to promote a shift toward M2 macrophages (91, 92). In rheumatoid arthritis, glucocorticoids similarly foster an anti-inflammatory macrophage phenotype, suppress cytokine production, boost phagocytic capacity, and help protect joint structures—partly via interactions with fibroblast-like synoviocytes (93). These effects also facilitate clearance of apoptotic cells, often through stromal cell interactions.

Classical immunosuppressants, while acting on a broad spectrum of immune cells, also impact monocyte and macrophage function through diverse mechanisms. Methotrexate induces apoptosis in monocytes and promotes the transformation of M1 macrophages into M2 macrophages through adenosine signaling. The levels of adenosine increase in response to methotrexate administration, binding to adenosine receptors on monocytes (94). In bleomycin-induced pulmonary fibrosis mouse models, tacrolimus inhibits JAK2/STAT3 signaling, reducing profibrotic factor production by M2 macrophages and modulating their polarization, suggesting a significant anti-fibrotic effect through macrophage targeting (95). However, tacrolimus can also promote M2-like polarization in monocytes/macrophages from healthy volunteers and inhibit p38MAPK phosphorylation at higher concentrations (96), indicating a complex and potentially concentration-dependent effect on macrophage function. Azathioprine’s metabolites, 6-MP and 6-T-GTP, dampen macrophage-mediated inflammation. While this effect is largely Rac1-independent, Rac1-mediated suppression of iNOS also contributes (97). Cyclophosphamide reduces the production of pro-inflammatory cytokines IL-1 and TNF by monocytes, acting both directly on these cells and indirectly through effects on lymphocytes and the hematopoietic system (98). Moreover, IVIg, though its mechanisms of action are complex, inhibits IFN-γ signaling in macrophages by suppressing the Fc receptor FcγRIII (99). Despite the ability of current immunosuppressants to reduce inflammation and modulate macrophage activity, they are often limited by side effects and may not fully resolve monocyte/macrophage-driven pathology. This underscores the need for more targeted therapeutic strategies aimed at specifically addressing the role of these cells in disease.

## Targeting monocyte recruitment and activation

Monocyte migration to inflamed tissues is a critical step in the amplification of inflammatory responses. Strategies designed to interfere with this process are of considerable interest. For instance, antibodies targeting the chemokine receptor CCR2, which is crucial for CCL2 (MCP-1)-mediated chemotaxis, have been explored as a therapeutic approach. While anti-CCR2 antibodies did not demonstrate substantial efficacy in rheumatoid arthritis trials (100), their potential in the context of IIMs, in which CCL2 is often elevated, deserves further consideration. Similarly, monoclonal antibodies targeting granulocyte-macrophage colony-stimulating factor, a key cytokine in monocyte and macrophage differentiation, are being explored for their ability to inhibit macrophage proliferation and activation (101). Although these antibodies are not currently under investigation specifically for IIM treatment, they are in clinical trials for treating other autoimmune conditions (e.g., rheumatoid arthritis, psoriasis, and multiple sclerosis) (101).

The colony-stimulating factor 1 receptor (CSF-1R) plays a crucial role in the survival, proliferation, differentiation, recruitment, and function of mononuclear phagocytes, including macrophages and monocytes (102). Therefore, monoclonal antibodies targeting CSF-1R are being actively investigated as well (103). A strategy to reduce monocyte and macrophage activation and recruitment to inflamed tissues involves targeting oxidative stress. In experimental autoimmune myositis models, increased ROS and decreased NF-E2-related factor 2 (Nrf2) levels are observed, and studies have shown that overexpressing Nrf2 in experimental autoimmune myositis macrophages inhibits their migration and reduces the levels of pro-inflammatory factors while increasing antioxidative stress enzymes (104). Hence, activating the Nrf2/antioxidant response element pathway can promote the degradation of ROS and reduce the expression of pro-inflammatory factors, thus potentially mitigating macrophage infiltration and inflammation in IIMs.

## Modulating macrophage polarization

The inherent plasticity of macrophages can allow a shift in their activation from a pro-inflammatory M1-like phenotype toward a reparative M2-like state. This concept is appealing, and several approaches to achieve this shift are under evaluation (105–107). However, as noted earlier, M2-like macrophages have also been implicated in IIM pathogenesis, particularly in the context of fibrosis. Since M2-like macrophages may be recruited in response to inflammation to aid in its resolution (108), it is important to recognize that the M2 phenotype is not monolithic. Indeed, M2 macrophages are further classified into subsets with distinct functions, including pro-fibrotic, immunomodulatory and anti-inflammatory, and immunosuppressive and pro-fibrotic roles (67). A shift toward anti-inflammatory and immunosuppressive subtypes may have therapeutic potential in IIM.

Modulating signaling molecules such as peroxisome proliferator-activated receptors (PPARs), particularly PPAR-γ, holds promise. PPAR-γ expression is increased by IL-4 and is a key regulator of inflammation and lipid metabolism, promoting an alternatively activated macrophage phenotype (109, 110). Mesenchymal stem cell-derived exosomes represent another avenue for modulating macrophage polarization. These vesicles can transfer diverse molecules, including DNA, mRNA, and proteins, to recipient cells, including macrophages, to promote tissue repair and suppress inflammatory M1 cells (111, 112). Additionally, exosomes derived from other cell types, such as endothelial cells (e.g., containing miR-10a) and adipose tissue-derived stem cells (e.g., carrying active STAT3, which induces arginase-1 expression in macrophages), can also modulate macrophage phenotypes (113, 114). As such, understanding which exosomes promote a more reparative macrophage phenotype is important for exploring mesenchymal stem cell-derived exosome therapy for IIMs.

## Targeting aberrant immune signaling and cellular dysfunction

The complex interplay between dysregulated mitochondrial function and heightened IFN signatures, especially in conditions like DM and JDM (1, 77, 115–119), highlights opportunities for intervention. Given that type I IFN signaling utilizes JAK1 and tyrosine kinase 2 (TYK2) as signal transducers, JAK inhibitors—some of which are already approved for rheumatoid arthritis (120) —show potential by blocking IFN-α and IFN-γ signaling, particularly in monocytes, as well as in other immune cells (121). Additionally, they promote the generation of monocyte-derived macrophages with an anti-inflammatory transcriptional and functional profile (122), thereby reducing the production of pro-inflammatory cytokines and chemokines. Indeed, some reports have shown the efficacy of JAK inhibitors, particularly in managing anti-MDA5 antibody-positive DM (123–131). This may stem from the robust type I IFN signature observed in anti-MDA5 antibody-positive DM. Furthermore, anifrolumab, a human monoclonal antibody targeting the type I IFN receptor subunit 1, which has been approved for SLE (132), is another potential therapeutic option. In addition to its broader effects on IFN signaling, anifrolumab has been shown to attenuate the inflammatory response in models of myocardial ischemia/reperfusion injury by reducing monocyte/macrophage polarization toward the pro-inflammatory M1 phenotype and decreasing their phagocytic activity (133). In fact, case reports have shown rapid improvement of skin rashes in IIM patients treated with anifrolumab (134, 135). Likewise, dazukibart, a monoclonal antibody against IFN-β, also showed promising results in a phase 2 trial for adult DM, especially for skin manifestations (136).

While direct targeting of inflammatory cytokines, such as TNF-α (137–142) and IL-6 (143, 144) is a therapeutic strategy being explored for IIMs, the efficacy of these agents varies and, in some cases, may cause harm (145–149). TNF-α inhibitors regulate the polarization of inflammatory M1 macrophages while also enhancing phagocytosis (150). Meanwhile, IL-6 inhibitors reduce superoxide anion production by monocytes/macrophages and upregulate PPAR-γ expression in monocytes and monocyte-derived macrophages, promoting a shift toward an anti-inflammatory phenotype (151). Further research is needed to identify and validate specific therapeutic targets, optimize drug use, and establish efficacy in well-defined IIM patient populations based on molecular data (152).

## Discussion

**Figure 1:**
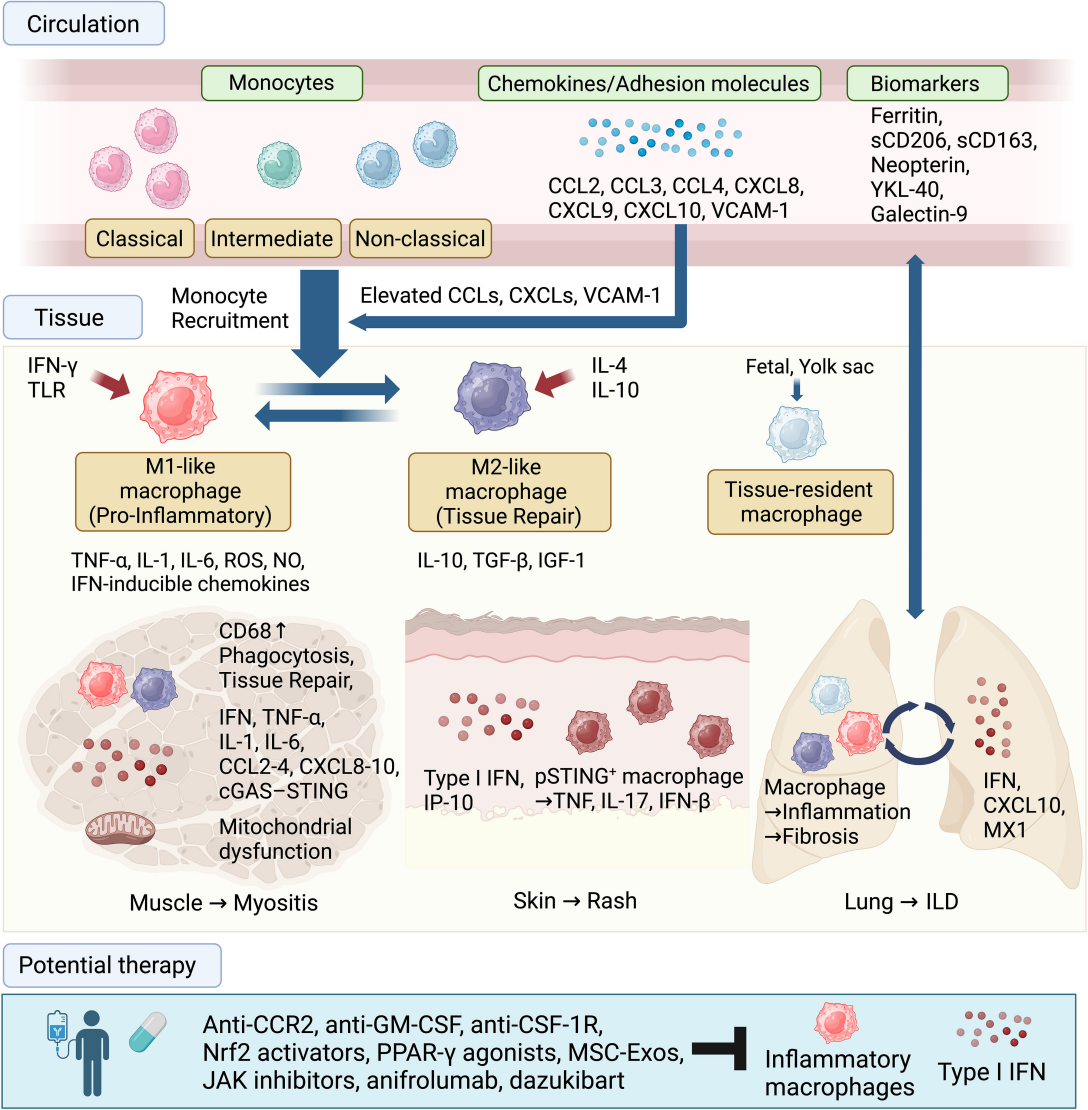
Involvement of monocytes and macrophages in IIMs. Monocytes from the bone marrow circulate in the blood (classical, intermediate, non-classical) and are recruited to affected tissues, such as muscle, skin, and lung, by elevated chemokines and adhesion molecules. In tissues, monocytes differentiate into pro-inflammatory (M1-like) macrophages or tissue-repairing (M2-like) macrophages under the influence of cytokines such as IFN-γ, IL-4, and IL-10. In the muscle, these macrophages contribute to phagocytosis and tissue damage, while in the lung, they are associated with interstitial lung disease (ILD), marked by elevated levels of ferritin and other biomarkers. Type I IFN responses are observed in affected organs. Potential therapeutic targets to address monocyte and macrophage involvement in IIMs include anti-CCR2, anti-GM-CSF, anti-CSF-1R, Nrf2 activators, PPAR-γ agonists, mesenchymal stem cell-derived exosomes (MSC-Exos), JAK inhibitors, anifrolumab, and dazukibart.

Monocytes and macrophages drive tissue inflammation, damage, and fibrosis in IIMs via complex interactions with innate and adaptive immunity (**Figure 1**). Their infiltration patterns differ according to subtype, reflecting distinct immunopathological pathways. Mitochondrial dysfunction and metabolic shifts regulate monocyte activation and cytokine production, complicating the traditional M1/M2 framework. In addition to therapies targeting various molecules and emerging technologies (153, 154), strategies are being developed to inhibit macrophage activation, shift them toward anti-inflammatory phenotypes, block key receptors (e.g., CSF-1R), or target their released cytokines.

Further studies are needed to clarify how autoantibody profiles and clinical phenotypes shape monocyte/macrophage phenotypes and clinical outcomes. Moreover, a recent large-scale retrospective cohort study revealed a higher risk of ischemic heart disease in PM and DM (hazard ratio: 1.61 [1.15–2.25]) (155). Given that one cause of atherosclerosis is the accumulation of cholesterol-laden macrophages in the arterial wall and that atherosclerotic plaques also contain monocytes (156), the increased risk of heart disease in IIM may be explained by another pathogenic role of monocytes and macrophages. The role of tissue-repairing macrophages in IIM remains poorly understood (86), and local tissue factors like hypoxia and muscle-derived signals also impact macrophage polarization. Advanced profiling methods (scRNA-seq, spatial transcriptomics) are vital for uncovering disease-specific subsets (157). Therefore, a personalized approach targeting dysregulated pathways of monocyte/macrophage biology in IIMs holds the potential to transform patient care, enabling mechanism-based therapies that balance inflammation, tissue repair, and fibrosis more effectively.

## Supplementary material

Supplementary Figure 1 Macrophage infiltration patterns in muscle across selected subtypes of IIM. Macrophage infiltration in the perimysium and endomysium among different dermatomyositis subtypes. TIF1-γ- and Mi-2-positive patients demonstrate the higher endomysial infiltration, whereas MDA5-positive patients show comparatively lower macrophage infiltration in both perimysial and endomysial areas. IIM, idiopathic inflammatory myopathies; MDA5, melanoma differentiation-associated gene 5; TIF1-γ, transcription intermediary factor 1-γ.
