# Supplementary figures and images for: The role of monocytes and macrophages in idiopathic inflammatory myopathies: insights into pathogenesis and potential targets
Source: Front Immunol. 2025 Mar 20;16:1567833. doi: 10.3389/fimmu.2025.1567833 (PMC11965591; doi:10.3389/fimmu.2025.1567833)

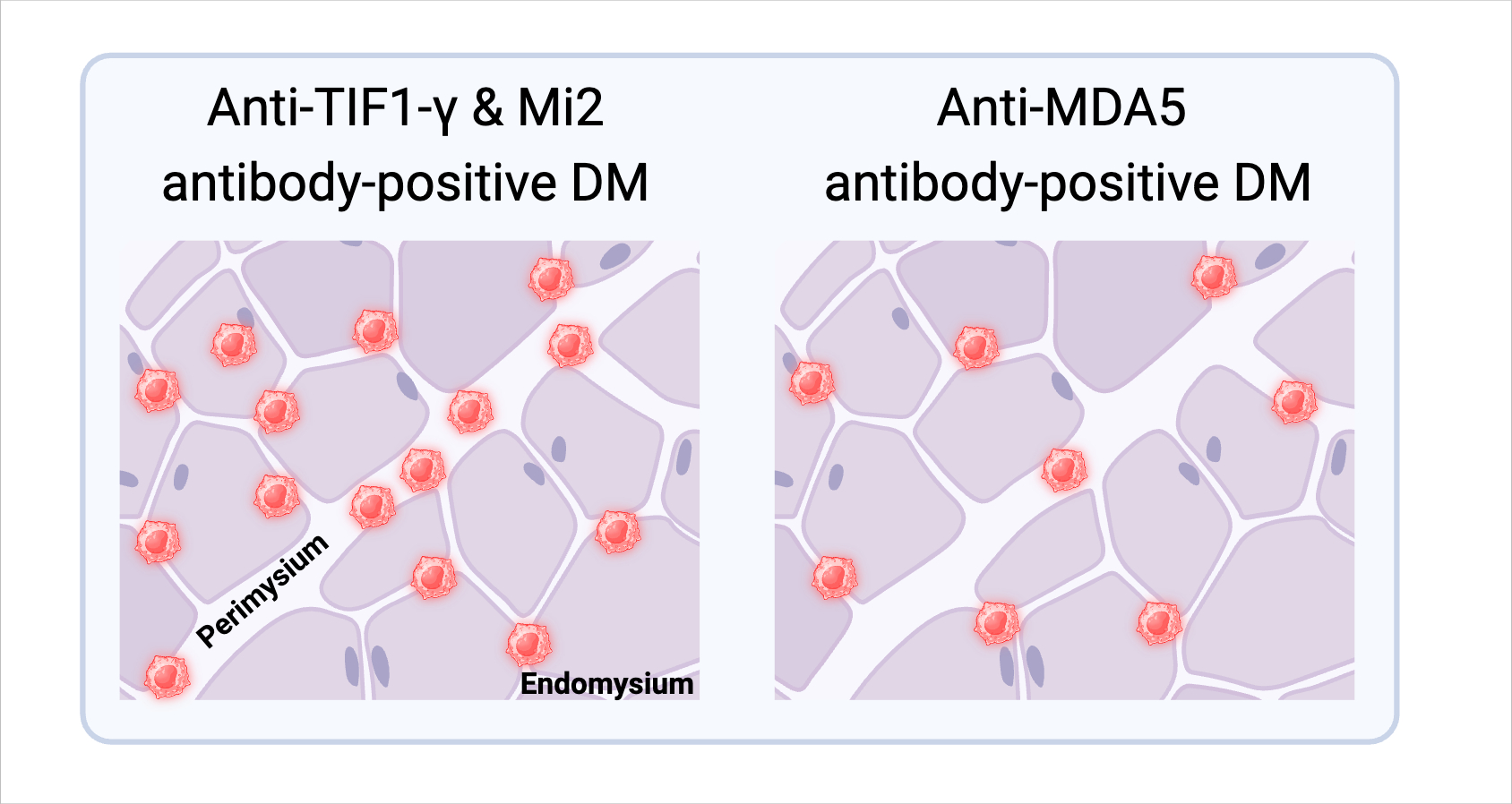

Supplement: Supplementary Figure 1 — Macrophage infiltration patterns in muscle across selected subtypes of IIM. Macrophage infiltration in the perimysium and endomysium among different dermatomyositis subtypes. TIF1-γ- and Mi-2-positive patients demonstrate the higher endomysial infiltration, whereas MDA5-positive patients show comparatively lower macrophage infiltration in both perimysial and endomysial areas. IIM, idiopathic inflammatory myopathies; MDA5, melanoma differentiation-associated gene 5; TIF1-γ, transcription intermediary factor 1-γ. [file Image1.jpeg]
